# Health Sunday

**Published:** 1916-02

**Authors:** 


### Health Sunday.

Hot Springs, the noted health resort, inaugurated the plan of having a physician fill every pulpit in the city one Sunady in the year to talk to the people on health problems. The suggestion might be followed profitably by every town. Only two objections could possibly be urged to this. Some of the over-religious might urge that the church is the place for preaching the gospel, but Christ taught the gospel of cleanliness and health about as forcefully as he urged the worship of God. Some physicians may fear that if the people are taught how to avoid sickness they will be out of employment, but those so narrow as this should be filling less responsible positions than practicing medicine.

